# Brain-derived neurotrophic factor: a bridge between inflammation and neuroplasticity

**DOI:** 10.3389/fncel.2014.00430

**Published:** 2014-12-22

**Authors:** Francesca Calabrese, Andrea C. Rossetti, Giorgio Racagni, Peter Gass, Marco A. Riva, Raffaella Molteni

**Affiliations:** ^1^Department of Pharmacological and Biomolecular Sciences, Università degli Studi di MilanoMilan, Italy; ^2^Department of Psychiatry and Psychotherapy, Research Group Animal Models in Psychiatry, Central Institute of Mental Health, Medical Faculty Mannheim/Heidelberg UniversityMannheim, Germany

**Keywords:** BDNF, neurogenesis, lipopolysaccaride, pro-inflammatory cytokines, depression

## Abstract

Cytokines are key regulatory mediators involved in the host response to immunological challenges, but also play a critical role in the communication between the immune and the central nervous system. For this, their expression in both systems is under a tight regulatory control. However, pathological conditions may lead to an overproduction of pro-inflammatory cytokines that may have a detrimental impact on central nervous system. In particular, they may damage neuronal structure and function leading to deficits of neuroplasticity, the ability of nervous system to perceive, respond and adapt to external or internal stimuli. In search of the mechanisms by which pro-inflammatory cytokines may affect this crucial brain capability, we will discuss one of the most interesting hypotheses: the involvement of the neurotrophin brain-derived neurotrophic factor (BDNF), which represents one of the major mediators of neuroplasticity.

## Neuroplasticity and brain-derived neurotrophic factor

For many years the medical field held the belief that the brain did not make major changes after a certain point in time. It was fixed or set on a specific path. Today, in contrast, we know that the brain is actually capable of changing and developing throughout a lifetime. It is plastic or malleable, and the term *neuroplasticity* is used to describe this tendency for the brain to keep developing, changing, and potentially healing itself.

Specifically, neuroplasticity or neuronal plasticity refers to the ability of the nervous system to respond and adapt to environmental challenges and encompasses a series of functional and structural mechanisms that may lead to neuronal remodeling, formation of novel synapses and birth of new neurons. Neuronal plasticity is intimately linked to cellular responsiveness and may therefore be considered an index of the neuronal capability to adapt its function to a different demand. Failure of such mechanisms might enhance the susceptibility to environmental challenges, such as stress, and ultimately lead to psychopathology.

Among the genes responsive to neuronal activity, neurotrophic factors (NTFs), and in particular the neurotrophin family, play an important role. In fact, besides their classical role in supporting neuronal survival, NTFs finely modulate all the crucial steps of network construction, from neuronal migration to experience-dependent refinement of local connections (Poo, 2001). These functions were first reported based on the observation that, during the development of the nervous system, neuron survival depends on the limited amount of specific NTFs secreted by target cells (Huang and Reichardt, 2001). However, it is now well established that NTFs are important mediators of neuronal plasticity also in adulthood where they modulate axonal and dendritic growth and remodeling, membrane receptor trafficking, neurotransmitter release, synapse formation and function (Lu et al., 2005). The neurotrophin brain-derived neurotrophic factor (BDNF) has emerged as crucial mediator of neuronal plasticity, since it is abundant in brain regions particularly relevant for plasticity, but also because it shows a remarkable activity-dependent regulation of expression and secretion (Bramham and Messaoudi, 2005), suggesting that it might indeed bridge experience with enduring change in neuronal function. BDNF has a complex genomic structure, which results into a sophisticated organization in terms of transcriptional, translational and post-translational regulatory mechanisms (Aid et al., 2007). In particular, the rat BDNF gene—that is similar to the human gene—can generate nine distinct transcripts through the alternative splicing of 5’ un-translated exons to a common 3’ exon (IX), which encodes the BDNF protein (Aid et al., 2007). These transcripts have different distribution and/or translation efficacy and, more importantly, may sub-serve different functions. For example, transcripts that are primarily localized or targeted to dendrites may sustain local neurotrophin production, thus providing an effective mechanism to regulate synaptic structure and function (An et al., 2008; Wu et al., 2011). Since the transcription of the different isoforms is regulated by specific signaling pathways (Pruunsild et al., 2011), their investigation may provide useful information on the up-stream mechanisms contributing to the changes of BDNF gene expression.

The mechanisms that lie downstream from NTFs and contribute to the maintenance of neuroplasticity are different i.e., adult neurogenesis and neuronal remodeling, but on the purpose of this mini-review we will focus only on adult neurogenesis, the process by which neurons are generated. Neurogenesis occurs under precise spatial and temporal control, but it can be modulated by both internal and external stimuli. Among these, several sources of data indicate the positive impact of BDNF on adult neurogenesis (Lee et al., 2002; Sairanen et al., 2005; Scharfman et al., 2005; Gass and Riva, 2007; Bergami et al., 2008; Chan et al., 2008; Li et al., 2008; Waterhouse et al., 2012), however in this review we will focus our attention on the effects of pro-inflammatory cytokines.

## Neurogenesis and inflammatory state

Neurogenesis has been defined as the process in which newborn neurons are generated from progenitors to functionally integrate in the neuronal network (Ming and Song, 2005; Balu and Lucki, 2009; Aimone et al., 2014). Actually, active neurogenesis take place, in the healthy central nervous system, only in two specific regions: neurons are continuously generated in the sub-ventricular zone (SVZ) and migrate into the olfactory bulb to become interneurons and, in parallel, neurogenesis occurs also in the sub-granular zone (SGZ) of the dentate gyrus of the hippocampus, where new granule neurons are continually generated. Depending on different stimuli, neural stem cells, located in so-called stem cell niches, could divide symmetrically, leading to the generation of two identical cells to maintain the pool of undifferentiated progenitors or, on the other hand, they can divide asymmetrically in order to generate an identical daughter cell and a second cell that starts to differentiate. The *de novo* formation and integration of new neurons into the existing circuitry is one of the various plastic changes that allow the adult brain to adapt to exogenous stimuli (Amrein et al., 2011). In particular, adult neurogenesis within the hippocampus could contribute to enhanced neural plasticity, a process that is fundamental for specific brain functions such as spatial learning, pattern discrimination, contextual memory and mood regulation (Clelland et al., 2009; Sahay et al., 2011; Denny et al., 2014). The important role of hippocampal neurogenesis is underlined by the fact that this system is altered after various types of negative stimuli such as stress, one of the major risk factors for psychiatric diseases. Specifically, repeated restraint and inescapable foot shock, two examples of physical stressors, inhibit one or more steps of adult neurogenesis in the dentate gyrus (Malberg and Duman, 2003; Pham et al., 2003); the social defeat paradigm leads to an inhibitory effect on cell proliferation and survival of newborn granule neurons in rodents (Czéh et al., 2002; Jun et al., 2012); and social isolation, which is associated with decreased neurogenesis and behavioral alterations in rodents, has been recently proven to be deleterious also for hippocampal neurogenesis and behavior in non human primates (Cinini et al., 2014).

As previously mentioned, neurogenesis is conditioned by a very complex microenvironment constituted by the vascular net, different growth and NTFs, changes in electrical and chemical environment and support by glial cells (Kohman and Rhodes, 2013). In this scenario, neuroinflammation is emerging as one of the main actors. In fact the immune system, through cells within the brain (e.g., microglia) and the detrimental or the beneficial action of signaling molecules (pro-inflammatory or anti-inflammatory cytokines) could participate in the response to different exogenous and endogenous stimuli. The negative effects of neuroinflammation on neurogenesis could lead to impaired survival and proliferation of new neurons. For example, intracortical or intraperitoneal administration of lipopolysaccaride (LPS) from *E. coli*, an agent able to induce a strong immune response, decreases new neurons survival and the differentiation of new cells into neurons (Ekdahl et al., 2003; Monje et al., 2003). The consequences of inflammation on neurogenesis could have also functional implications for cognition. In fact, the impact of neuroinflammation could affect also the correct integration of newborn neurons into pre-existing circuits, through changes in cellular morphology and in electrophysiological properties (Jakubs et al., 2008) and reduction in recruitment into hippocampal networks encoding spatial information (Belarbi et al., 2012).

## The impact of pro-inflammatory cytokines on neurogenesis

Neuroinflammation has an important role in the pathophysiology of different acute or chronic CNS disorders such as cerebral ischemia, multiple sclerosis, Alzheimer’s disease, Parkinson’s disease and major depression (Wang and Jin, 2014). These diseases are characterized by the modulation of different mediators of inflammation and among them pro-inflammatory cytokines seem to play a key role. It is important to note that the same cytokines that in a physiological state are involved in the maintenance of neuronal integrity, may instead have detrimental effects under pathological conditions. Accordingly, the impact of the pro-inflammatory cytokines on neurogenesis depends on their concentration, on the specific cells activated (astrocytes and microglia) and on the presence of other factors secreted in the neurogenic niche (Eyre and Baune, 2012). The increase of pro-inflammatory cytokines is not only due to a direct inflammatory stimulus (infection or trauma), but it could be caused by environmental stimuli such as stress (García-Bueno et al., 2008). The main consequence of a dysregulation of cytokine levels within the brain is the production of inflammatory, oxidative and nitrosative molecules that could affect neurogenesis and the neural homeostasis (Kubera et al., 2011; Stepanichev et al., 2014).

The most common pro-inflammatory cytokines are IL-1β, IL-6, TNF-α and IFN-γ and here we will present some examples of the involvement of these molecules in the modulation of neurogenesis.

The main actions of IL-1β are the stimulation of immune cells to produce pro-inflammatory cytokines, the activation of microglia, and the regulation of growth factors activity (Audet and Anisman, 2013). Recently, IL-1β has been proven to influence hippocampal cytogenesis and neurogenesis in different ways: by direct interaction to its receptor (IL-1R1) and the consequent activation of the nuclear factor-kappa B (NFkB; Koo and Duman, 2008) or through the promotion of glucocorticoids secretion after the exposure to environmental stressors (Goshen et al., 2008). Moreover this cytokine has been proposed as the central mediator of antineurogenic effect of stress (Ben Menachem-Zidon et al., 2008). In fact the blockade of IL-1β signaling, using knockout mice for its receptor or administrating a IL-1R1 antagonist (IL-1Ra), prevents the decrease in neurogenesis observed after acute stressors such as footshock and immobilization in rats (Koo and Duman, 2008). Another relevant cytokine is IL-6 that is involved in a multitude of neuroprotective functions. In physiological conditions IL-6 is able to activate pathways related to neural plasticity, neurogenesis, Long Term Potentiation, and memory (Eyre and Baune, 2012). On the other hand, this cytokine is also responsible of mediating synthesis of acute phase proteins, growth and differentiation of immune cells and regulation of pro-inflammatory factors (Audet and Anisman, 2013). Monje et al. demonstrated that the incubation of hippocampal progenitor cells with recombinant IL-6 decreases neurogenesis by half and reduces neuronal differentiation in favor of astrocytogenesis (Monje et al., 2003; Taga and Fukuda, 2005), an effect mediated by the activation of the JAK/STAT3 pathway via gp130 (Namihira and Nakashima, 2013). Tumor necrosis factor—alpha (TNF-α) is a potent inductor of inflammation and has been linked to decreased neural stem cell proliferation, decreased neurogenesis, neurodegenerative processes, apoptosis and excitotoxicity (Dantzer et al., 2008; Belarbi et al., 2012), but also to the modulation of synaptic strength and synaptic preservation through the increase of the α-amino-3-hydroxy-5-methyl-4-isoxazolepropionic acid (AMPA) receptors (Khairova et al., 2009). The negative action of TNF-α on neurogenesis is mediated by the activation of its receptor TNF-R1, conversely the interaction with TNF-R2 increases proliferation and survival of newborn neurons, as demonstrated by using transgenic animals with deletion of TNF-R1 or TNF-R2 (Iosif et al., 2006). A similar result on the beneficial role of TNF-R2 activation after irradiation injury has been recently reported (Chen and Palmer, 2013). Moreover, the up-regulation of TNF-α observed in the hippocampus of adult rats pre-exposed to maternal deprivation has been associated with impaired memory consolidation (Pinheiro et al., 2014). IFN-γ is a pro-inflammatory cytokine with *in vitro* anti-neurogenic effect able to reduce the number of neural stem cells. The negative action of IFN-γ on neurogenesis may be exerted by the activation of the caspase 3/7, the upregulation of sonic hedgehog (SHH) pathway and promotion of an abnormal marker profile of neural stem cells, expressing both GFAP and βIII tubulin (Walter et al., 2011). Nevertheless, IFN-γ may also exerts positive action on neurogenesis. For example, it enhances neurogenesis in dentate gyrus of adult mice and ameliorates spatial learning and memory performance (Baron et al., 2008). These observations suggest that IFN-γ has different effects depending on tissues involved and on the neurogenic process involved.

Taken together, all these studies indicate that a dysregulation of pro-inflammatory cytokines may have a detrimental effect on neurogenesis and point out the importance of neuroinflammation in the microenvironment around neural stem cell development. On this context, the identification and characterization of the mechanisms by which pro-inflammatory cytokines affect neurogenesis are crucial to develop new strategies to maintain the proper function of stem cell niches within the brain.

## The impact of pro-inflammatory cytokines on BDNF

Given the role of BDNF as an important mediator of neuroplasticity and on the basis of its positive contribution on neurogenesis in contrast to the detrimental effect of pro-inflammatory cytokines, we may hypothesize that one of the mechanisms by which inflammation may affect brain function could involve BDNF modulation.

Several *in vivo* studies demonstrated that inflammation clearly affects the expression of BDNF within the brain. In particular, it has been reported that the administration of pro-inflammatory cytokines or of the cytokine-inducer lipopolysaccharide, (LPS; Raetz and Whitfield, 2002) causes a significant reduction of BDNF gene expression. For example, the mRNA levels of BDNF were significantly decreased in the rat hippocampus 4 h after intraperitoneal injection of IL-1β or LPS (Lapchak et al., 1993) and a similar reduction was also observed in several cortical regions and at protein level (Guan and Fang, 2006; Schnydrig et al., 2007). Interestingly, the effect of the systemic inflammatory challenge was not restricted to BDNF: other neurotrophins such as nerve growth factor (NGF) and neurotrophin-3 (NT-3) were similarly reduced although with different magnitude (Guan and Fang, 2006).

Recently, it has also been evaluated the effect of peripheral immune challenge on the different BDNF transcripts, finding that the expression of exons I, II, and IV in the dentate gyrus was reduced in the CA1 and in the dentate gyrus of rats acutely treated with *E. coli* (Chapman et al., 2012), indicating that inflammation may affect specific isoforms of the neurotrophin. Nevertheless, there is a critical lack of information about the effects of inflammation on the expression of specific BDNF transcripts and further studies are demanded in order to clarify the mechanisms involved in the modulation of the neurotrophin by the immune/inflammatory system.

The negative impact of inflammation on BDNF has important implications for a number of pathological conditions. For example, it is known that pro-inflammatory cytokines compromise hippocampus-dependent memory (Pugh et al., 1998), spatial memory (Arai et al., 2001) and increase apoptosis in the brain (Nolan et al., 2003), features that are involved in many aging-associated pathologies and neurodegenerative diseases. In addition, it is well-know that the activation of the immune/inflammatory system may contribute to the development of different psychiatric diseases such as schizophrenia and major depression (Dantzer et al., 2008; Miller et al., 2009; Leonard and Maes, 2012; Zunszain et al., 2013). Regarding depression, there are three main supportive evidences: first, depressed subjects exhibit increased levels of inflammatory markers both in the periphery and in brain (Howren et al., 2009; Dowlati et al., 2010); second: several pathologies associated with moderate inflammatory grade present high depression comorbidity (Benton et al., 2007); third: a high percentage of patients with cancer or hepatitis C treated with interferon-alpha develop major depression (Valentine and Meyers, 2005; Udina et al., 2012). In addition, it has to be noted that animals exposed to immune challenges display depressive-like behaviors (Yirmiya, 1996; Frenois et al., 2007) that can be normalized, or at least limited, by antidepressant treatment (Yirmiya et al., 2001). In contrast, mice that lack IL-6 are stress resistant and have a reduced disposition for depressive-like behaviors (Chourbaji et al., 2006). The mechanism underlying the anti-inflammatory properties of antidepressant is still unknown and is beyond the aim of our mini-review. However, the results of several *in vitro* and *in vivo* studies indicate that these drugs are able to modulate cytokine functioning through their effects on intracellular cyclic adenosyl monophosphate, serotonin metabolism, the hypothalamo-pituitary-adrenocortical axis (Janssen et al., 2010; Walker, 2013; Leonard, 2014).

**Figure 1:**
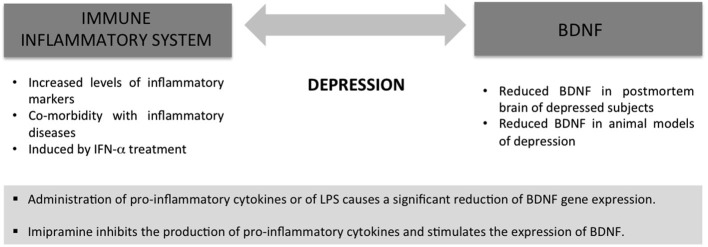
**Immune/inflammatory alterations and changes in BDNF expression and function are characteristic of the same pathologies, for example major depression, suggesting a cross-talk between the two systems**.

It is important to consider that the immune/inflammatory alterations previously described are actually in parallel with changes on BDNF expression and function (Figure 1). Indeed, BDNF has a well recognized role in the etiology as well as in the treatment response of patients affected by different psychiatric disorders including major depression (Pezet and Malcangio, 2004; Duman and Monteggia, 2006). For example, decreased expression of the neurotrophin has been found in the hippocampus and prefrontal cortex of postmortem brains from depressed and suicide victims (Dwivedi et al., 2003). Moreover, BDNF mRNA levels are reduced in the brain of genetic animal models of depression (Ridder et al., 2005; Calabrese et al., 2010; Molteni et al., 2010a,c) as well as in animal models based on the environmental component of the disease (Duman and Monteggia, 2006; Tsankova et al., 2006; Chourbaji et al., 2012).

All these findings support the possibility that inflammation contributes to the development of depression by compromising neuroplasticity via reduction of BDNF. In agreement with this line of thinking, it has been recently reported that intranigral LPS infusion induced an anxious and depressive phenotype in the rat that was associated with decreased hippocampal expression of BDNF (Hritcu and Gorgan, 2014).

In order to have a unequivocal proof for causality, inflammation-dependent decrease of BDNF should be normalized or at least attenuated by antidepressant treatment, as occurs in experimental models where BDNF expression is up-regulated in response to prolonged treatment with different antidepressant drugs (Schmidt and Duman, 2007; Calabrese et al., 2010; Molteni et al., 2010b; Park et al., 2011).

Although there are only few data on this issue, it has been demonstrated that the incubation of rat neural stem cells with the antidepressant imipramine inhibits the production of pro-inflammatory cytokines, whereas stimulates the expression of BDNF (Peng et al., 2008), nevertheless, further studies are demanded to clarify this issue in order to provide unequivocal proof for causality.

## Concluding remarks

**Figure 2:**
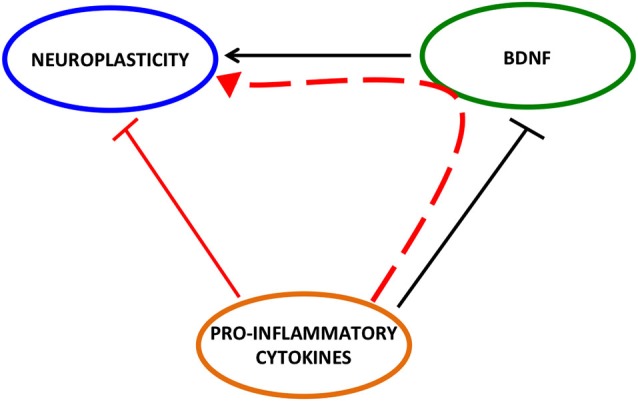
**Detrimental effect of pro-inflammatory cytokines on neuroplasticity may be mediated by BDNF**.

In conclusion, we attempted to provide evidence on the possibility that one of the mechanisms underlying the negative impact of pro-inflammatory cytokines on neuroplasticity is the reduction of BDNF expression and function (Figure 2).

Although several data support this hypothesis, further studies are demanded to better clarify how it occurs. A number of result points out a key role for the pro-inflammatory cytokine IL-1β as it has been shown that the inhibitory effect of stress paradigms on cerebral BDNF expression may be attenuated by intracerebroventricular injection of IL-1 receptor antagonist (Barrientos et al., 2003). However, how this -or others- pro-inflammatory cytokine affects the neurotrophin is still not well understood. Since *in vitro* and *in vivo* studies indicate that glucocorticoids decrease the neurotrophin (Hansson et al., 2003; Gubba et al., 2004; Hansson and Fuxe, 2008), one possibility is the involvement of the Hypotalamus-Pituitary-Axis (HPA), which is strongly stimulated by pro-inflammatory cytokines (Rivest, 2010). However, we have to be aware that pro-inflammatory cytokines act on a plethora of different targets, for example the neurotransmitters glutamate (Viviani et al., 2007; Di Filippo et al., 2013) and GABA (Galic et al., 2012), both able to modulate BDNF. In this context, it is feasible that the effect of the immune/inflammatory system on BDNF results from the integration of multiple mechanisms. A better knowledge of these events may be useful to develop new therapeutic strategies aimed to normalize, or at least ameliorate, the pathological consequences of the negative impact of inflammation on brain structure and function.

## Conflict of interest statement

The authors declare that the research was conducted in the absence of any commercial or financial relationships that could be construed as a potential conflict of interest.
